# Brainstem Modulation of Large-Scale Intrinsic Cortical Activity Correlations

**DOI:** 10.3389/fnhum.2019.00340

**Published:** 2019-10-09

**Authors:** R. L. van den Brink, T. Pfeffer, T. H. Donner

**Affiliations:** ^1^Department of Neurophysiology and Pathophysiology, University Medical Center Hamburg-Eppendorf, Hamburg, Germany; ^2^Department of Psychology, University of Amsterdam, Amsterdam, Netherlands; ^3^Amsterdam Center for Brain and Cognition, Institute for Interdisciplinary Studies, Amsterdam, Netherlands

**Keywords:** functional connectivity, norepinepherine, dopamine, acetycholine, serotonin, brainstem, neuromodulation, resting-state

## Abstract

Brain activity fluctuates continuously, even in the absence of changes in sensory input or motor output. These intrinsic activity fluctuations are correlated across brain regions and are spatially organized in macroscale networks. Variations in the strength, topography, and topology of correlated activity occur over time, and unfold upon a backbone of long-range anatomical connections. Subcortical neuromodulatory systems send widespread ascending projections to the cortex, and are thus ideally situated to shape the temporal and spatial structure of intrinsic correlations. These systems are also the targets of the pharmacological treatment of major neurological and psychiatric disorders, such as Parkinson’s disease, depression, and schizophrenia. Here, we review recent work that has investigated how neuromodulatory systems shape correlations of intrinsic fluctuations of large-scale cortical activity. We discuss studies in the human, monkey, and rodent brain, with a focus on non-invasive recordings of human brain activity. We provide a structured but selective overview of this work and distil a number of emerging principles. Future efforts to chart the effect of specific neuromodulators and, in particular, specific receptors, on intrinsic correlations may help identify shared or antagonistic principles between different neuromodulatory systems. Such principles can inform models of healthy brain function and may provide an important reference for understanding altered cortical dynamics that are evident in neurological and psychiatric disorders, potentially paving the way for mechanistically inspired biomarkers and individualized treatments of these disorders.

## Introduction

Neural population activity in the cerebral cortex fluctuates continuously, even in the absence of changes in sensory input or motor output. These so-called intrinsic cortical activity fluctuations show remarkable structure across time and space: activity fluctuations correlate across sets of distributed brain areas, on the basis of which macroscale functional networks can be delineated (Biswal et al., 1995; Fox and Raichle, 2007). Such intrinsic activity correlations are commonly studied in a setting that is often referred to as the “resting state”: the absence of motor output or structured sensory input (often with eyes-closed). However, intrinsic activity fluctuations that correlate across time and space also occur during active processing of sustained, unchanging, sensory input (Donner et al., 2013; Meindertsma et al., 2017; Pfeffer et al., 2018). We therefore use the term “intrinsic activity correlations,” as it is agnostic about the behavioral context.

We focus on intrinsic correlations between cortical population signals that pool the activity across thousands of individual neurons. Intrinsic activity fluctuations have also been investigated at the level of single-neuron spiking, in this context commonly referred to as “noise correlations” (Zohary et al., 1994; Cohen and Kohn, 2011; Nienborg et al., 2012; Kohn et al., 2016). Similar to the intrinsic correlations between cortical population signals reviewed below, noise correlations between single neurons are state-dependent (e.g., Harris and Thiele, 2011; Reimer et al., 2014; Joshi and Gold, 2019). An important open question, beyond the scope of this review, is whether or not the same mechanistic principles account for the impact of state variations on neural correlations at these different (microscopic vs. macroscopic) scales. In this article, we use the term “intrinsic (cortical activity) correlations” to exclusively refer to correlations between cortical population signals.

Although predominantly studied with functional magnetic resonance imaging (fMRI), intrinsic correlations have also been shown to occur using electro-/magnetoencephalography (E/MEG), electrocorticography, and other imaging modalities (Friston et al., 1993; Mao et al., 2001; Nir et al., 2008; de Pasquale et al., 2010; Hipp et al., 2012; Lewis et al., 2016; Siems et al., 2016; Stitt et al., 2018; Hollensteiner et al., 2019) and show spatiotemporal correspondence across modalities (Hipp and Siegel, 2015; Siems et al., 2016). The temporal structure of intrinsic activity varies across cortical areas, which may reflect inter-areal variation in computational properties such as intrinsic timescales (Murray et al., 2014) that depend on inter-areal projections (Chaudhuri et al., 2015) and, possibly, functional interactions (Baria et al., 2013). Moreover, intrinsic activity correlations are largely predictive of task-related activation patterns (Cole et al., 2016; Tavor et al., 2016), and provide useful diagnostic and prognostic markers of neurological and psychiatric disorders (Fox and Greicius, 2010; van den Brink et al., 2018b). Thus, intrinsic activity correlations are a ubiquitous phenomenon and their quantitative features (Box 1) are potentially revealing indicators of the functional architecture of the brain.

**Box 1. Quantifying features of intrinsic activity correlations.**

In this article, we discuss three characteristics of intrinsic activity correlations:

• The *strength/magnitude* of correlations in activity. Correlation strength can be used to examine the extent to which activity between any two brain regions is correlated (so-called seed-based correlation analysis), or to examine the overall “connectedness” of the brain by averaging the correlation coefficient across all brain region or deriving summary statistics such as “degree” (Rubinov and Sporns, 2010) or “functional connectivity density” (Tomasi and Volkow, 2010).
• *Topography:* A spatial representation of (some property of) a system. For example, the spatial distribution of a particular resting state network (RSN), defined as a set of brain regions or voxels that shows consistent spatio-temporal dynamics (usually above a particular threshold). The topography is often used to characterize the structure of individual RSNs, or experimental manipulation-related changes therein (Smith et al., 2009).
• *Topology*: The geometrical relationship between elements of a system. The human brain has been argued to approximate a *small world* topology, which forms a mixture of dense connections between neighboring brain regions and sparse long-range connections (Watts and Strogatz, 1998; Sporns and Zwi, 2004; Bassett and Bullmore, 2006; Bassett et al., 2006). Such a topology allows for both distributed and integrated processing, the balance between which relates to task-performance (Shine et al., 2016; Shine and Poldrack, 2018).

A number of observations indicate that the features of intrinsic activity correlations are shaped by the architecture of anatomical connections of the cerebral cortex. First, intrinsic correlations within the visual system reflect established principles of cortico-cortical projections, such as retinotopic organization (Heinzle et al., 2011; Donner et al., 2013; Gravel et al., 2014; Bock et al., 2015). Second, computational models that are equipped with realistic anatomical connectivity can predict the topological features and temporal dynamics of empirical intrinsic correlations reasonably well (Honey et al., 2007, 2009; Cabral et al., 2012). Third, causal manipulation of anatomical connections alters the strength of functional interactions (O’Reilly et al., 2013). This line of inquiry thus suggests that the full repertoire of functional interactions across the brain is shaped by the anatomical substrate upon which these interactions unfold (Deco et al., 2011).

However, other observations indicate that the anatomical connectome alone is not *sufficient* to account for the features of intrinsic activity correlations. The correspondence between the anatomical and functional connectome varies with attentional (Baria et al., 2013) and conscious (Barttfeld et al., 2015) state, and shows substantial temporal variability even within periods of rest (Chang and Glover, 2010; Sakoglu et al., 2010; Allen et al., 2014; Zalesky et al., 2014; Lurie et al., 2018). What are the sources of these variations of intrinsic activity correlations?

**FIGURE 1:**
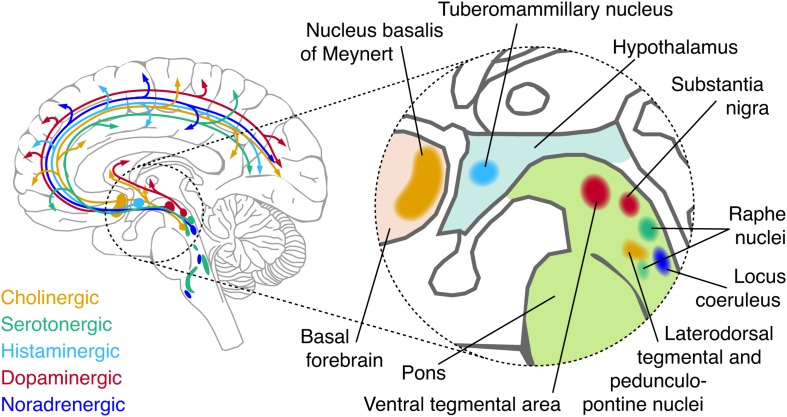
Schematic of major neuromodulatory systems. Cerebellar, spinal, and temporal projections are omitted for brevity. The inset shows the approximate anatomical location of each nucleus that sends major afferents to the forebrain.

Here, we focus on one candidate source that has received surprisingly little attention in the resting-state literature, but, as we propose, is crucial for understanding the origin, dynamics, and diagnostic value of intrinsic activity correlation: the neuromodulatory systems of the brainstem. The term refers to a small set of brainstem nuclei with widespread projections to the forebrain, which synthesize and release specific modulatory neurotransmitters (“neuromodulators”; Figure 1 and Box 2). By virtue of their widespread projection profiles and effects on the state of cortical target networks, these systems can shape neural activity across the cortex in a coordinated fashion. Consequently, these systems are in an ideal position to shape intrinsic activity correlations. What is more, these brainstem systems are disturbed in several major psychiatric disorders, which also coincide with changes in intrinsic activity correlations (Calhoun et al., 2009; Rosazza and Minati, 2011; Wang et al., 2012; Vargas et al., 2013; Baggio et al., 2015; Dichter et al., 2015; Mulders et al., 2015; Giraldo-Chica and Woodward, 2017).

**Box 2. Major brainstem neuromodulatory systems.**

Besides various peptides, five major neuromodulatory systems have been identified:

• *Norepinephrine* (NE) is released by the locus coeruleus (LC), and the A1/A2 regions of the brainstem (Sara, 2009). The LC projects to virtually all areas of the forebrain with the exception of the basal ganglia. Projection specificity of sub-populations of LC neurons has recently been shown (Chandler and Waterhouse, 2012; Chandler et al., 2014; Schwarz and Luo, 2015; Schwarz et al., 2015; Uematsu et al., 2015, 2017; Rho et al., 2018; Cerpa et al., 2019).
• *Dopamine* (DA) is predominantly released by two nuclei: the substantia nigra pars compacta (SNpc) and the ventral tegmental area (VTA) (Foote and Morrison, 1987). Four major dopaminergic branches exist of which three are ascending: the mesolimbic (VTA to ventral striatum), mesocortical (VTA to cortex) and nigrostriatal (SNpc to dorsal striatum) pathways. In addition, DA co-release by the LC (Takeuchi et al., 2016; Beas et al., 2018) and serotonergic dorsal raphe nuclei (Cho et al., 2017) have recently been shown. Other DA-producing neurons are found in the olfactory bulb (Pignatelli and Belluzzi, 2017) and pedunculopontine nucleus (French and Muthusamy, 2018). DA and NE have a similar chemical composition and are collectively known as catecholamines.
• *Acetylcholine* (ACh) is released by neurons in the basal forebrain (BF), which is comprised of several subdivisions, termed Ch1-Ch4, that contain cholinergic neurons. Ch4 corresponds to the nucleus basalis of Meynert, and is the major source of cortical ACh (Mesulam and van Hoesen, 1976; Mesulam et al., 1983; Mesulam and Changiz, 1988). This nucleus contains topographically organized clusters of neurons that preferentially innervate select portions of the cortex (Rho et al., 2018; Zaborszky et al., 2018; Ahmed et al., 2019). Other sources of ACh include the pedunculopontine nucleus and laterodorsal tegmental nucleus of the brainstem, which project to the thalamus, basal ganglia, hypothalums, and cortex (Statoh and Fibiger, 1986; Garcia-Rill, 1991; French and Muthusamy, 2018).
• *Serotonin* (5HT) originates from the raphe nuclei, which is a constellation of nuclei scattered throughout the brainstem (Törk, 1990). The raphe nuclei can be roughly subdivided in rostral and caudal portions, of which the rostral portion can be further subdivided in a dorsal (B6-B7) and median (B5/B8) portion. Both the dorsal rand median raphe project heavily to the cortex (Törk, 1990).
• *Histamine* is released by the tuberomammilary nucleus of the hypothalamus that projects to virtually the entire forebrain (Haas and Panula, 2003).

Most neuromodulatory brainstem nuclei do not consist of a single type of neuron releasing one neuromodulator, but contain a mixture of multiple types of neurons that can include GABAergic or glutamatergic types as well as other neuromodulators (Lin et al., 2015; Cho et al., 2017; Beas et al., 2018; Breton-Provencher and Sur, 2019). Furthermore, some of these nuclei are reciprocally connected and their activity tends to co-fluctuate in unison with changes in arousal and wakefulness (Foote and Morrison, 1987; Haas and Panula, 2003; Sara, 2009).

In what follows, we review the dependence of intrinsic activity correlations on neuromodulatory systems. An increasing number of studies over the past decade, conducted in humans, macaques, and rodents (rats and mice), have begun to provide insight into this dependence. Our goal is to provide a structured overview of this nascent literature and distil from it a number of emerging principles. This article is a selective review, which focuses on emerging principles rather than a comprehensive coverage of the literature. Moreover, we focus on the neuromodulatory systems on which most work has been conducted. In particular, the catecholaminergic systems (norepinephrine, NE; and dopamine, DA) were among the first systems to be studied in relation to intrinsic activity correlations. This article also covers more recent work into the acetylcholine (ACh) and serotonin (5HT) systems. To date, comparatively little work has been conducted on the system-level effects of histamine, and it will therefore not be discussed in the current review.

## Candidate Mechanisms of Brainstem Modulation of Intrinsic Cortical Activity Correlations

The distinction between two modes of cortical state provides a useful heuristic for conceptualizing potential effects of brainstem neuromodulatory systems on cortical network dynamics: “*activity state*” and “*dynamic state*” (Curto et al., 2009; Safaai et al., 2015). This (likely oversimplified) dichotomy can be formalized by means of dynamical systems models. Curto et al. (2009) and Safaai et al. (2015) used the FitzHugh–Nagumo model [originally developed for describing action potential generation (FitzHugh, 1961; Nagumo et al., 1962)] for modeling cortical population dynamics (Curto et al., 2009; Safaai et al., 2015). In this framework, *activity state* refers to the set of parameters that vary on timescales from milliseconds to hundreds of milliseconds, and *dynamic state* refers to the set of parameters that vary more slowly (from seconds to tens of seconds) and *interact multiplicatively with* (i.e., modulate) the fast variations of activity states (Curto et al., 2009; Luczak et al., 2009; Harris and Thiele, 2011; Safaai et al., 2015).

In physiological terms, activity state can be conceptualized as common measures of “neuronal activity”: membrane potential or spiking activity. Changes in these measures of neuronal activity are caused by excitatory or inhibitory postsynaptic potentials, mediated by point-by-point synaptic transmission via ionotropic receptors (predominantly for glutamate and GABA). This form of synaptic transmission is the means of intracortical interactions and lies at the heart of current large-scale computational models of intrinsic activity correlations (Honey et al., 2007, 2009; Breakspear et al., 2009; Deco et al., 2013, 2014).

By contrast, variations in dynamic state can be conceptualized as the slower effects of neuromodulators, mediated by “volume transmission” (i.e., not point-by-point synapses) and by metabotropic receptors that do not alter the postsynaptic membrane potential directly. Activation of metabotropic receptors sets in motion intracellular signaling cascades that alter the way in which neurons respond to input over protracted periods of time, from changing the conductance of ionotropic receptors to altering the expression of genes. For example, catecholamines (in particular noradrenaline) change the balance between excitation and inhibition in the local microcircuit (Froemke, 2015; Martins and Froemke, 2015; Pfeffer et al., 2018). This circuit effects in turn increases the responsivity of cortical neurons to synaptic input (Moises et al., 1979; Rogawksi and Aghajanian, 1980; Seamans et al., 2001a, b; Wang and O’Donnell, 2001), an effect referred to as “neural gain”: an increase in the slope of the input-output function (Berridge and Waterhouse, 2003; Murphy and Miller, 2003; Winterer and Weinberger, 2004; Disney et al., 2007; Polack et al., 2013). This mechanism of action corresponds to the common notion of “neuromodulation.”

Importantly, while the majority of receptors for neuromodulators are metabotropic, some of them are ionotropic – in particular, the nicotinic class of ACh receptors (Itier and Bertrand, 2001), and the 5HT_3_ subclass of serotonin receptors (Barnes et al., 2009). Activation of both receptor types leads to rapid activation of cortical networks (Puig et al., 2004; Fu et al., 2014; McCormick and Nusbaum, 2014; Wester and McBain, 2014; McGinley et al., 2015). Moreover, changes in the activity of neuromodulatory nuclei coincide with (Eschenko et al., 2012), and cause (Pinto et al., 2013) rapid fluctuations in activity state (e.g., the transition from the “down” to “up” state of synchronized cortical population activity). Serotonergic or cholinergic activation of ionotropic receptors thus constitutes a mechanism by which neuromodulatory brainstem system can rapidly change the cortical activity state.

In sum, neuromodulators may, in principle, alter intrinsic activity correlations in two ways. First, by rapidly changing the activity state of distributed sets of cortical regions through common (excitatory or inhibitory) drive (Drew et al., 2008). Second, neuromodulators may change the dynamic state (e.g., excitation–inhibition balance) of sets of cortical regions in a coordinated fashion but on slower timescales. Such coordinated changes in dynamic state, in turn, may directly produce correlations between population activity, but they can also modulate the correlations produced by cortical interactions through altering local dynamics (Deco et al., 2014; Pfeffer et al., 2018). The two principal mechanisms (modulation of activity state vs. dynamic state) reflect the distinct effects of cortical (ionotropic vs. metabotropic) receptors. Importantly, both mechanisms can produce intrinsic activity correlations (Leopold et al., 2003), even in the absence of any effect on cortico–cortical interactions.

**FIGURE 2:**
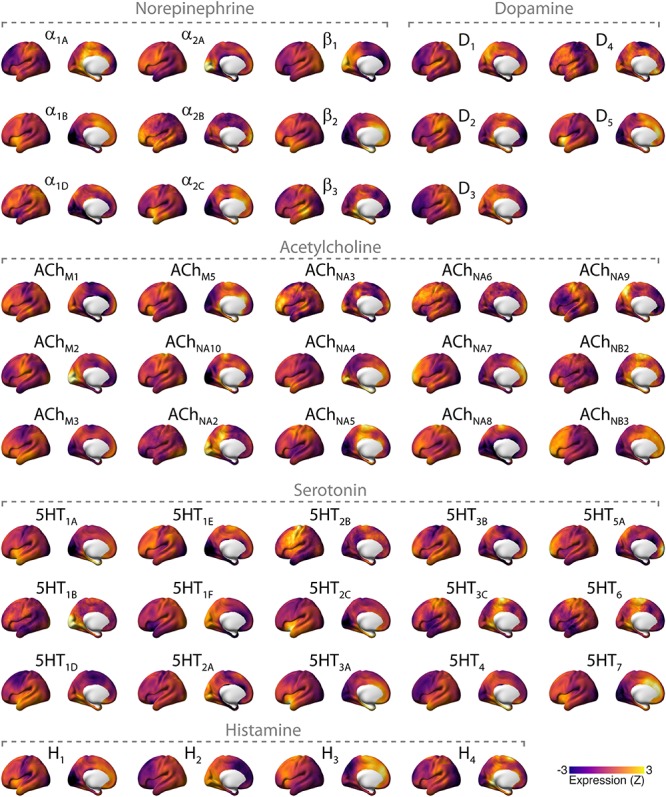
Overview of cortical distributions of genetic expression of neuromodulator receptors in the human brain. Receptor maps were taken from Gryglewski et al. (2018), projected onto the cortical surface, and Z-scored across space. Files and tool for plotting these maps can be found here: github.com/rudyvdbrink/receptormaps. Abbreviations: D: dopamine; ACh: acetylcholine; M: muscarinic; N: nicotinic; 5HT: serotonin; H: histamine.

While the projections of neuromodulatory nuclei to the cortex are commonly known as widespread or diffuse, there is substantial heterogeneity and specificity in these projections, part of which is only now being uncovered through novel anatomical tracing techniques (Foote and Morrison, 1987; Chandler and Waterhouse, 2012; Chandler et al., 2014; Schwarz and Luo, 2015; Schwarz et al., 2015; Uematsu et al., 2015, 2017; Kebschull et al., 2016; Breton-Provencher and Sur, 2019). What is more, the cortical distributions of the various different receptors for each neuromodulator are heterogeneous (Ramos and Arnsten, 2007; Zilles and Amunts, 2009; Nahimi et al., 2015; Salgado et al., 2016), which is evident for the human cortex in recent maps of receptor gene expression (Figure 2). Consequently, input from any neuromodulatory nucleus to the cortex, changing activity state, dynamic state, or both, might translate into spatially structured correlations of neural population signals in the cortex. For this reason, it is critical to consider the potential impact of neuromodulatory brainstem systems when making inferences about physiological cortico–cortical interactions (and “cortical networks”) from the correlation of intrinsic cortical activity alone.

## Correlated Cortical Activity Driven by Intrinsic Fluctuations of Brainstem Activity

If neuromodulatory nuclei rapidly drive cortical activity in widespread target networks via ionotropic mechanisms then (i) removing neuromodulatory drive on the cortex should attenuate correlated activity within the cortex, and (ii) manipulating the time-varying activity of neuromodulatory nuclei should similarly affect the time-varying activity within the cortex, and (iii) activity within neuromodulatory nuclei should co-vary with intrinsic activity in large areas of the cortex. These predictions also hold if neuromodulatory nuclei potentiate drive from other sources such as the thalamus.

### Causal Manipulation of Time-Varying Neuromodulatory Activity

Evidence for the first two predictions comes from studies in which the time varying activity of neuromodulatory nuclei is manipulated. Turchi et al. (2018) reversibly inactivated portions of the BF using the GABA_A_ agonist muscimol in rhesus macaques, thus removing potential fluctuating common input to cortical areas. This manipulation reduced correlations between the “global” BOLD-fMRI signal (i.e., the average across all gray matter voxels) and voxel-wise activity topographically aligned with the afferents of the inactivated BF location. Because the BF sends GABAergic as well as cholinergic projections to the cortex, these findings do not necessarily reflect cholinergic effects. Grandjean et al. (2019) rhythmically stimulated serotonergic neurons in the DR with optogenetics in rodents, and measured the cerebral blood volume (CBV) response with fMRI (CBV was used because of putative signal-to-noise advantages over BOLD-fMRI). Cortical CBV showed widespread, correlated troughs in amplitude in response to DR stimulation. Moreover, the cortical distribution of CBV responses correlated spatially with reductions in burst rate and delta-band power as measured electrophysiologically.

Combined, these two studies (Turchi et al., 2018; Grandjean et al., 2019) provide the strongest evidence to date of a mediation of (a part of) intrinsic activity correlations through rapid, common input from neuromodulatory brainstem nuclei that are consistent with correlated changes in activity state. A critical test of this notion would, however, use as a neural marker of interest correlations between spiking activity in different cortical regions, rather than between their BOLD or CBV signals. This is because changes in the latter signals may not necessarily reflect changes in cortical activity state (i.e., spiking activity; Maier et al., 2008) but may be produced by changes in dynamic state (Logothetis, 2008).

Analogous evidence for the LC-NE system comes from older positron emission tomography (PET) work by Coull et al. (1999) who reduced LC-activity via clonidine (Florin-Lechner et al., 1996) in healthy humans. During rest, clonidine caused broad reductions in directed coupling of PET activity between several cortical and sub-cortical regions. Due to the sluggish nature of the PET signals measured, however, it is difficult to attribute these results to changes in activity or dynamic state. In addition, DA depletion has similarly been reported to cause broad reductions in fMRI activity correlation strength (Shafiei et al., 2019).

### Temporal Co-variation Between the Brainstem and the Cortex

The prediction that activity in neuromodulatory nuclei should co-vary with cortical activity can be tested using seed-based correlation analyses, where (fMRI) activity in neuromodulatory nuclei is correlated with cortical activity. A limited number of such studies have been conducted. Because of the small size and spatial proximity of these nuclei to the ventricles (Figure 1) and strong effects of physiological noise, these types of measurements require non-standard approaches to fMRI measurement and data analysis (Astafiev et al., 2010; Klein-Flugge et al., 2011; Brooks et al., 2013; Beissner, 2015; de Gee et al., 2017; Forstmann et al., 2017). Therefore, unless mentioned otherwise, we only considered studies where retrospective image correction for physiological noise was applied.

Zhang et al. (2015) assessed correlations between the cortex and LC, VTA, and SN, as defined by anatomical atlases (Ahsan et al., 2007; Keren et al., 2009). They reported widespread negative correlations between the cortex and all three brainstem nuclei and predominantly positive correlations between activity in these nuclei and other subcortical areas. Liu et al. (2018) found that peaks in the global (gray matter voxel averaged) signal coincided with troughs in activity of the BF, suggesting an anti-correlation between BF activity and cortical activity. This is in line with earlier findings by Li et al. (2014), who reported widespread negative correlation between BF and cortical areas, although no correction for physiological noise was applied in this study. By contrast, Markello et al. (2018) found positive correlations between anatomically defined BF subdivisions (Zaborszky et al., 2008) and known cortical targets of BF projections, possibly due to the fact that in this study partial correlation was used to examine subdivision-specific correlations. Lastly, Beliveau et al. (2015) found relatively confined partial correlations between the cortex and anatomically and [^11^C]DASB PET-binding constrained delineations of the dorsal and median raphe nuclei.

### Summary and Outstanding Issues

The most direct evidence for intrinsic activity correlations through common drive of distributed cortical regions by neuromodulatory systems comes from direct manipulations of the activity of the corresponding brainstem nuclei. However, such studies are sparse and have not yet been conducted for all neuromodulatory nuclei. In addition, activity in most, but not all, neuromodulatory nuclei has been reported to covary with widespread areas of the cortex. However, to the best of our knowledge, no study to date has examined the individual contribution of the full set of neuromodulatory nuclei (Figure 1 and Box 2) to intrinsic activity fluctuations within the cortex. This leaves open the possibility that any correlation between an individual nucleus and the cortex is due to shared fluctuations across neuromodulatory nuclei (de Gee et al., 2017) rather than reflecting specific drive of one neuromodulatory nucleus on cortical activity. Moreover, removal of the global signal (as done by e.g., Beliveau et al., 2015) may obscure wide-spread correlations and reveal only those correlations that are stronger than global components of the cortical signal (i.e., the mean of all cortical regions). Another important limitation of all studies assessing intrinsic activity correlations by means of the fMRI signal, is that that the latter may reflect either changes in activity state (spike rate) or changes in dynamic state produced by neuromodulatory mechanisms (Logothetis, 2008).

In sum, the available studies are consistent with the notion of common drive of distributed cortical regions by the fluctuating activity of neuromodulatory nuclei. In this light, at least part of the spatial structure of intrinsic activity correlations within the cortex may reflect the spatial distribution of the projections of these brainstem nuclei, or their receptors, rather than the topography of cortico-cortical connections. Further experiments and direct comparisons of the contribution of individual neuromodulatory nuclei on intrinsic activity correlations are warranted, bearing in mind the above-mentioned interpretational caveats.

## Changes in Intrinsic Cortical Correlations Under Pharmacological Intervention

A major approach in the study of neuromodulatory systems is to manipulate neuromodulator levels via pharmacological intervention, and measure the resulting effects on cortical activity. Such pharmacological intervention will primarily exert its effects on cortical activity through sustained alterations of cortical circuit properties (i.e., shifts in cortical dynamic state), and less (or not at all) by altering the rapid drive of cortical regions, although it has been shown that the NE-reuptake inhibitor atomoxetine alters the dynamics of LC activity (e.g., via auto-receptors) (Bari and Aston-Jones, 2013). For simplicity, we here heuristically treat pharmacological intervention as manipulations of changes in cortical dynamic state, and review the effects of these manipulations on intrinsic activity correlations.

We examine three key characteristics of these correlations (Box 1) in order to delineate commonalities or inconsistencies across findings. First, given the widespread projection profile of neuromodulatory systems (Figure 1 and Box 2), pharmacological manipulation of these systems may be expected to result in changes in correlation strength that encompass large areas of the cortex. We thus discuss literature that has examined such global changes of correlation strength (i.e., whole-brain increases or decreases). Second, the spatial heterogeneity of neuromodulatory projections and receptors (Figure 2) across the cortex suggests that neuromodulatory systems may not only change the global strength of intrinsic activity correlations, but also result in spatially inhomogeneous changes of correlation strength (van den Brink et al., 2018a). Third, computational modeling work indicates that modifications of circuit properties that are subject to neuromodulatory influence such as gain or excitation–inhibition balance (Servan-Schreiber et al., 1990; Polack et al., 2013; Froemke, 2015; Pfeffer et al., 2018) can alter the geometric properties of whole-brain intrinsic activity correlations (topology), even without any heterogeneity of neuromodulatory influences (Deco et al., 2014; Shine et al., 2018a). We therefore discuss literature that has examined the effect of pharmacological manipulation of neuromodulator systems on RSN topography and whole-brain functional topology.

### Changes of the Global Strength of Intrinsic Correlations

Several studies have examined the effect of the NE reuptake inhibitor atomoxetine, which increases cortical catecholamine levels (Bymaster et al., 2002; Devoto et al., 2004; Swanson et al., 2006; Koda et al., 2010), on global correlation strength. Using atomoxetine, van den Brink et al. (2016) found reductions compared to placebo in graph-theoretic metrics of the global strength of intrinsic fMRI activity correlations in humans. Similarly, Guedj et al. (2017b) found reduced atomoxetine-induced fMRI correlation strength within and between various RSNs in rhesus macaques, with an overall net change of reduced correlation. This was also reflected in a reduction in the average brain-wide weighted correlation coefficient (Guedj et al., 2017a). By contrast, Pfeffer et al. (2019) found no significant atomoxetine-induced change in graph theoretic metrics of activity correlations measured with MEG during rest. However, during viewing of a perceptually ambiguous visual stimulus task, Pfeffer et al. (2019) found that atomoxetine *increased* the strength of correlations. A global increase in fMRI correlations following chemogenetic LC stimulation in anesthetized mice has also been reported (Zerbi et al., 2019).

The above mentioned MEG-study by Pfeffer et al. (2019) also investigated the effect of increased cortical ACh levels on correlated activity. This study used the acetylcholinesterase inhibitor donepezil and found reduced correlation strength during rest, and no effect during the presentation of an ambiguous visual stimulus. Three studies have examined the effect of pharmacological modulation of the 5HT system on the global strength of fMRI correlations. Schaefer et al. (2014) reported widespread reductions in correlation strength (quantified as the graph theoretic metric degree) following administration of the 5HT reuptake inhibitor escitalopram. Similarly, Preller et al. (2018) found a widespread shift of cortical intrinsic activity correlations toward zero due to the 5HT_2__A_ receptor agonist LSD – an effect that did not occur when simultaneously administering the 5HT_2__A_ antagonist ketanserin. By contrast, Tagliazucchi et al. (2016) reported an LSD-induced increase in the overall strength of correlations within the cortex.

### Topographically Specific Changes of Intrinsic Correlation Strength

Topographical effects of pharmacological manipulation may be informative about which sets of cortical regions (“networks”) are particularly susceptible to neuromodulatory influence through a modulation of the dynamic state. Below, we provide a summary of consistent findings in the literature (see also Tables 1, 2). We review studies that either used whole-brain “dual regression,” or correlation between a seed and the entire cortex. The term dual regression refers to sequential spatial and temporal regression of independent components with the purpose of identifying consistent spatiotemporal networks and manipulation-related changes therein (Beckmann, 2009).

**TABLE 1:** Summary of main findings of (pharmacological) manipulation of catecholamines (NE and DA).

**Study**	**Modality(species)**	**Analysismethod**	**Manipulation**	**Main effects of manipulation on intrinsic correlations**
Coull et al., 1999	PET (humans)	Dynamic causal modeling	Clonidine (α2 agonist)	Rest: reduced effective connectivity from PFC to thalamus, and to and from visual cortex. Attentional task: general increase in effective connectivity, with changes centered on parietal cortex.
Achard and Bullmore, 2007	fMRI (humans)	Graph theoretic analysis	Sulpride (D_2_ antagonist)	Reduced metrics of global and local efficiency.
Kelly et al., 2009	fMRI (humans)	Seed-based correlation	L-DOPA (DA precursor)	Increased correlation between putamen and cerebellum and brainstem. Increased correlation between ventral striatum and vlPFC. Reduced correlation between DMN, and ventral striatum and caudate.
McCabe and Mishor, 2011	fMRI (humans)	Seed-based correlation	Reboxetine (SNRI)	Reboxetine: reduced amygdala-OFC correlation, and reduced striatal–OFC correlation.
Cole et al., 2013	fMRI (humans)	Dual regression	Haloperidol (D_2_ antagonist) L-DOPA (DA precursor)	Linear increase (haloperidol < placebo < L-DOPA) between BG network and sensorimotor cortex. Inerted-U between BG network and dorsal anterior-mid cingulate (placebo > haloperidol and L-DOPA). Linear decrease (haloperidol > placebo > L-DOPA) between DMN and sensorimotor cortex. Linear increase (haloperidol < placebo < L-DOPA) between DMN and SMG.
Akeju et al., 2016	fMRI (humans)	Dual regression Seed-based correlation	Dexmedetomidine (α2 agonist)	Reduced correlation between DMN and bilateral thalamus and left cerebellum; increased correlation between DMN and IFG, putamen, and insula. Reduced correlation between rlFPN and cerebellum; increased correlation between rlFPN and cerebellum, precuneus, parietal operculum insula fusiform and angular gyri. Reduced correlation between llFPN and cerebellum, calcarine cortex, MFG, and SFG.
Metzger et al., 2015	fMRI (humans)	Seed-based correlation	Reboxetine (SNRI) Amisulpride (D_2/3_ antagonist)	Reboxetine: increased correlation between brainstem, and thalamus and PCC; and thalamus and accumbens; reduced correlation between putamen-brainstem; amygdala-ACC; and between the accumens and two regions of the ACC; reduced correlation between accumbens and two regions in ACC. Amisulpride: increased correlation between: PCC-brainstem; amygdala, brainstem and thalamus; putamen-brainstem.
van den Brink et al., 2016	fMRI (humans)	Graph theoretic analysis Seed-based correlation	Atomoxetine (SNRI)	Reduced metrics of global correlation strength and clustering. Reduced correlation between FPN, and DMN and visual network, and reduced correlation between the visual and sensorimotor network. Reduced correlation within a set of occipital regions. Reduced correlation between early visual cortex and the rest of the brain.
Guedj et al., 2017a	fMRI (rhesus macaque)	Dual regression	Atomoxetine (SNRI)	Reduced correlation within FPN, somatosensory, sensorimotor, visual, and superior temporal sulcus networks. Reduced correlation between various networks with an overall net change of reduced correlation.
Guedj et al., 2017b	fMRI (rhesus macaque)	Graph-theoretic analysis	Atomoxetine (SNRI)	Reduced global efficiency. Reduced global correlation strength. Increased clustering.
Ye et al., 2017	fMRI (humans)	Graph theoretic analysis and seed-based correlation	Pramipexole (D_2_ agonist)	Reduced correlation between caudate and nodes of the sensorimotor network. No change in topological metrics.
Shine et al., 2018b	fMRI (humans)	Graph theoretic analysis	Atomoxetine (SNRI)	Rest: reduced metrics of integration. N-back task: increased metrics of integration.
van den Brink et al., 2018a	fMRI (humans)	Generalized eigenvalue decomposition	Atomoxetine (SNRI)	Increased correlations in a network that loosely resembled a rlFPN, and distribution of β (negatively) and D_2_ receptors. Reduced correlations in a network that loosely resembled a llFPN, sensorimotor and DMN networks, and distribution of β (positively) and α_1_ receptors.
Pfeffer et al., 2018	MEG (humans)	DFA + computational model	Atomoxetine (SNRI)	Rest: reduced α scaling exponent due to atomoxetine. Task (bistable perception): reduced α scaling exponent due to atomoxetine. Computational model accounted for the findings as a change in excitation relative to inhibition.
Shafiei et al., 2019	fMRI (humans)	Seed-based correlation Graph theoretic analysis	Phenylalanine and tyrosine depletion (DA depletion)	Reduced correlations strength in sensorimotor, salience, and temporal networks. Reduced between-module correlation of the sensorimotor and salience networks.
Pfeffer et al., 2019	MEG (humans)	Graph theoretic analysis + computational model	Atomoxetine (SNRI)	Rest: no effect of atomoxetine. Task (bistable perception): Atomoxetine increased correlation strength. Computational models indicate that effects can be accounted for by an increase in gain.
Zerbi et al., 2019	fMRI (mice)	Graph theoretic analysis (FCD) Dual regression	Chemogenetic LC stimulation	Increase in global correlation strength. Increased correlation in the Salience, amygdala, auditory, striato-motor, and DMN networks.

*ACC: anterior cingulate cortex; BG: basal ganglia; DA: dopamine; DFA: detrended fluctuation analysis; DMN: default mode network; vmPFC: ventromedial prefrontal cortex; LC: locus coeruleus; ll: left lateralized; MFG: middle frontal gyrus; IFG: Inferior frontal gyrus; vlPFC: ventrolateral prefrontal cortex; NE: norepinephrine; OFC: orbitofrontal cortex; SMG: supramarginal gyrus; PCC: posterior cingulate cortex; rl: right lateralized; FPN: frontoparietal network; SFG: superior frontal gyrus; SNRI: selective norepinephrine reuptake inhibitor.*

**TABLE 2:** Summary of main findings of (pharmacological) manipulation of 5HT and Ach.

**Study**	**Modality(species)**	**Analysismethod**	**Manipulation**	**Main effects of manipulation on intrinsic correlations**
Tanabe et al., 2011	fMRI (humans)	ICA-based back-reconstruction	Nicotine (nACh receptor agonist)	Reduced correlations within the DMN. Increased correlations within extrastriate cortex.
McCabe and Mishor, 2011	fMRI (humans)	Seed-based correlation	Citalopram (SSRI)	Citalopram: reduced amygdala-vmPFC correlation.
McCabe et al., 2011	fMRI (humans)	Seed-based correlation	Citalopram (SSRI)	Reduced correlation between left dmPFC and left hippocampus.
van de Ven et al., 2013	fMRI (humans)	Dual regression	Escitalopram (SSRI)	Reduced DMN correlations with PCC, ACC, hippocampus, and lateral parietal cortex.
Schaefer et al., 2014	fMRI (humans)	Graph theoretic analysis (FCD)	Escitalopram (SSRI)	Reduced global strength of correlation. Local increases in thalamus and cerebellum.
Klaassens et al., 2015	fMRI (humans)	Dual regression	Sertraline (SSRI)	Widespread decreases in correlation with DMN; executive control; visual and sensorimotor networks. Increased correlation between auditory network and PCC/precuneus.
Carhart-Harris et al., 2016	fMRI (humans)	Seed-based correlation	LSD (5HT agonist)	Increase in correlation between visual cortex and widespread regions of the cortex and between the parahippocampal cortex and the retrosplenial cortex and PCC, and increased correlations between parahippocampal cortex and dorsal mPFC and right dorsolateral PFC.
Tagliazucchi et al., 2016	fMRI (humans)	Graph theoretic analysis. Seed-based correlation	LSD (5HT agonist)	Increased global correlation strength. Brain regions that showed altered correlation strength overlapped with 5HT_2A_-r distributions. Increased correlation between four seeds (PFC, parietal cortex, precuneus, and thalamus) and sensorimotor areas. Reduced modularity. Increased participation coefficient of frontal and midline regions. Reduced rich-club coefficient.
Klaassens et al., 2017	fMRI (humans)	Dual regression	Citalopram (SSRI) Galantamine (nACh receptor agonist)	Citalopram: reduction of correlations within the sensorimotor network, PFN, DMN, and executive control network. Galantamine: increased correlations between polar occipital network and distributed regions; between an auditory network and regions of the DMN and somatosensory cortex; reduced correlations within the DMN, and between DMN and lateral and inferior occipital cortices; between the FPN and DMN, inferior temporal and cerebellar regions.
Deco et al., 2018	fMRI (humans)	Dynamic FC + computational model	LSD (5HT agonist)	The model captured the effect of 5HT_2A_-r stimulation as an increase in gain to explain the effect of LSD on the distribution of FC dynamics.
Turchi et al., 2018	fMRI (rhesus macaque)	Global signal correlation and dual regression	Pharmacological inactivation of basal forebrain	Broad reductions in coupling of local activity with the global signal, corresponding spatially to the inactivated location. Topography of individual RSNs unaffected.
Lord et al., 2018	fMRI (humans)	Dynamic FC	Psilocybin (5HT agonist)	Longer dwell times for a global FC component. Reduced dwell times for FPN.
Preller et al., 2018	fMRI (humans)	Graph theoretic analysis (FCD) Seed-based correlation	LSD (5HT agonist)	Reduced correlation (from positive toward zero) in associative networks. Increased correlation (from negative toward zero) in sensorimotor and thalamic networks. LSD effects correlated with 5HT_2A_-r distributions. Reduced correlation between sensorimotor areas and global signal.
Pfeffer et al., 2018	MEG (humans)	DFA + computational model	Donepezil (acetylcholinesterase inhibitor)	No effect of donepezil.
Pfeffer et al., 2019	MEG (humans)	Graph theoretic analysis + computational model	Donepezil (acetylcholinesterase inhibitor)	Rest: reduced correlation strength. Computational models indicate that effect can be accounted for by an increase in gain.
Grandjean et al., 2019	fMRI, MUA, and LFP (mice)	GLM analysis	Blockwise optogenetic stimulation of DR	Wide suppression of cortical CBV response. Suppression of cortical MUA, and δ LFP power, which spatially correlated with the cortical CBV response. CBV response correlated with distribution of 5HT 1F, 2A, 2C receptors, but not 1A and 1B receptors. Correlations with receptor maps were stronger than correlation with DR projection profile.

*5HT: serotonin; ACh: acetylcholine; ACC: anterior cingulate cortex; CBV: cerebral blood volume; DMN: default mode network; dmPFC: dorsomedial prefrontal cortex; DR: dorsal raphe; FC: functional connectivity; FPN: frontoparietal network; FCD: Functional connectivity density; PCC: posterior cingulate cortex; nACh: nicotinic acetylcholine; PCA: principle component analysis; SSRI: selective serotonin reuptake inhibitor.*

Pharmacological elevations of the noradrenergic tone, while yielding a diversity of findings, have consistently produced effects that involve visual cortex. Coull et al. (1999) reported an α_2_ agonist clonidine-induced reduction in effective connectivity to- and from posterior extrastriate visual cortex. Reduced correlations between early visual cortex and frontoparietal cortical areas have also been reported following the α_2_ agonist dexmedetomidine (Akeju et al., 2016) and NET blocker atomoxetine (van den Brink et al., 2016). Similarly, Guedj et al. (2017b) reported atomoxetine-induced reductions in correlation within a frontoparietal network and peripheral visual network. One study did not report changes in correlation of visual cortical areas following chemogenetic LC stimulation in rodents (Zerbi et al., 2019), potentially due to inter-species differences or differences between the effect of chemogenetic and pharmacological manipulation (Giorgi et al., 2017).

Manipulations of the cholinergic system have, likewise, yielded topographical effects that involve visual cortex. Tanabe et al. (2011) reported a nicotine-induced increase in correlation strength within an extrastriate network, and Klaassens et al. (2017) reported a galantamine (cholinesterase inhibitor) induced increase in correlation strength between a polar occipital network and widespread areas of the cortex. In addition, some, but not all (Guedj et al., 2017b), studies using noradrenergic or cholinergic agents have reported effects that involve the default mode network (DMN) (Tanabe et al., 2011; Akeju et al., 2016; van den Brink et al., 2016, 2018a; Klaassens et al., 2017; Zerbi et al., 2019). The observation that noradrenergic or cholinergic agents consistently produced effects on intrinsic correlations in visual cortical areas could be due to the fact that NE receptors α_2__A_ and β_1_, and ACh receptors NA_10_ and M_2_ are prominently expressed these regions (Figure 2).

In contrast, primarily dopaminergic agents produced no effects on visual cortex, but instead on somatosensory and (pre-)motor cortex. For example, a positive relationship between DA levels and correlation strength between the basal ganglia and sensorimotor cortex (Cole et al., 2013), reduced correlation between the caudate and nodes of the sensorimotor network including pre- and postcentral gyri following D_2_ receptor agonism (Ye et al., 2017), and reduced correlation within the sensorimotor network following DA depletion (Shafiei et al., 2019). Dopaminergic effects in motor cortical regions may reflect direct modulations of dynamic state within the cortex, or down-stream effects of modulations of the efficacy of dopaminergic projections from the brainstem to the basal ganglia (Figure 1).

Catecholaminergic manipulations have also been studied in the context of stress-related (re)activation patterns (Hermans et al., 2011; Gerlicher et al., 2018). These studies show prominent noradrenergic and dopaminergic effects on stress-induced changes in activation patterns or later reemergence thereof.

Studies using serotonergic agents have reported effects on intrinsic correlations resembling a combination of noradrenergic/cholinergic and dopaminergic effects: in other words, effects in both visual cortical and sensorimotor areas (Klaassens et al., 2015, 2017; Carhart-Harris et al., 2016; Tagliazucchi et al., 2016; Preller et al., 2018). In particular, studies that used the 5HT_2__A_ receptor agonist LSD consistently reported effects on visual cortex. Indeed, the 5HT_2__A_ receptor is prominently expressed in visual cortex (Figure 2).

### Topologically Specific Changes of Intrinsic Correlation Strength

Various analytical tools exist to characterize the topology of functional brain organization (Rubinov and Sporns, 2010; Shine and Poldrack, 2018). For instance, the balance between topological segregation and integration of cortical ensembles is determined by the ratio of activity correlation strength within versus between separate modules (Mattar et al., 2015; Shine et al., 2016), and has been related to behavioral performance (Shine and Poldrack, 2018). Topological variations in the segregation-integration balance of fMRI activity during rest covary with pupil diameter (Shine et al., 2016), a non-invasive proxy for activity in neuromodulatory nuclei (Murphy et al., 2014; Varazzani et al., 2015; Joshi et al., 2016; Breton-Provencher and Sur, 2019). Thus, topological features of intrinsic activity correlations may be under neuromodulatory control.

Indeed, pharmacological upregulation of cortical NE levels using atomoxetine has been shown to result in a shift toward segregated processing during rest, and a converse shift toward integrated processing during the performance of a cognitively demanding (N-back) task (Shine et al., 2018b). Similarly, van den Brink et al. (2016) found that atomoxetine reduced between-module correlation strength, and reduced metrics of integration (clustering coefficient and transitivity) during rest. Guedj et al. (2017a) reported reduced global efficiency, a metric of integration (Rubinov and Sporns, 2010), but reduced clustering, due to atomoxetine in rhesus macaques.

Studies using DA manipulations seem to indicate that DA facilitates integration. Achard and Bullmore (2007) reported reduced metrics of global and local efficiency due to the D_2_ antagonist sulpride. Shafiei et al. (2019) reported that DA depletion reduced the participation coefficient (between-module correlation) of the sensorimotor and salience networks. Thus, DA and NE may have dichotomous effects on network topology. However, null effects of DA agonism on various topological metrics, including metrics of integration, have also been reported (Ye et al., 2017). To the best of our knowledge, no studies to date have examined the effect of ACh manipulation on network topology, and one study has examined the effect of 5HT_2__A_ agonism (via LSD) on fMRI intrinsic correlation topology (Tagliazucchi et al., 2016). This study reported reduced modularity (increased integration), increased participation coefficient of frontal and midline regions (increased between-network correlation at the expense of within-network correlation), and reduced rich-club coefficient (less correlation with hub regions).

### Summary and Outstanding Issues

Pharmacological manipulation of tonic neuromodulatory action, and the resulting, putative change in cortical dynamic state, consistently alters the global strength of intrinsic correlations. Less consistent, however, is the direction of these effects, even *within* classes of neuromodulators. Further studies are needed to corroborate or exclude the following possible reasons for these discrepancies: cross-study differences in preprocessing such as global signal regression (Preller et al., 2018); dose-dependence of effects (Zahrt et al., 1997); or dependence of effects on cognitive/behavioral context (Coull et al., 1999; Shine et al., 2018b; Pfeffer et al., 2019) or baseline arousal (Warren et al., 2016).

Topographical effects following noradrenergic or cholinergic manipulation consistently involve visual cortex. Studies that used predominantly dopaminergic agents consistently report effects involving motor-related brain areas, but not visual brain areas. Studies that used a serotonergic agent report both visual and motor areas. This literature suggests that the regions that are most likely to be affected by pharmacological manipulation of neuromodulators are potentially distributed in accordance with the distribution of receptors across areas.

The predominant finding from studies on topological effects is that neuromodulators alter functional network topology, in particularly the catecholamines. These studies also suggest a possible distinction between the effects of DA and NE on network-level integration. Whereas NE reuptake during rest reduces topological metrics of integration, DA antagonism and depletion have the same effect, suggesting that DA facilitates integration. Similar to DA, 5HT seems to increase metrics of integration, but only one study has examined these effects. The influence of ACh on functional network topology remains to be studied.

A caveat with the findings on network topology is that some metrics of integration (in particular efficiency and clustering) are susceptible to changes in degree, even when artificially fixing degree of adjacency matrices by applying a fixed threshold (van Wijk et al., 2010). Since neuromodulators have been reported to alter degree as well (see section *changes of the global strength of intrinsic correlations*), future studies should carefully consider alterations in global degree when examining topological metrics. In addition, studies that have examined changes in the time-varying topology should take into account the influence of temporal fluctuations of the community structure on topological metrics (Thompson et al., 2019).

## Conclusion and Future Directions

Both (de)activation studies and seed-based correlation studies provide supporting evidence for ongoing fluctuations in the activity of neuromodulatory brainstem nuclei as a possible driving source of intrinsic activity correlations within the cortex. Temporary BF inactivation, NE release inhibition, DA synthesis inhibition, and rhythmic optogenetic serotonergic DR neuron stimulation all reduce intrinsic activity correlations. Furthermore, activity fluctuations in most neuromodulatory nuclei (BF; VTA; SN; LC) but not all (raphe nuclei) predict correlated activity fluctuations in broad areas of the cortex.

Pharmacological manipulation of cortical neuromodulator levels, which putatively alters cortical dynamic state, results in diverse changes of intrinsic activity correlations. First, pharmacological upregulation consistently changes the global strength of cortical correlations. Yet, in what direction the individual modulators exert their effects needs further study, since these effects are likely to be dependent on several factors, such as drug dose or behavioral context.

Second, several pharmacological studies have quantified neuromodulator-induced changes in the topography of cortical activity. Noradrenergic and cholinergic manipulation consistently alter activity correlations in visual cortical areas, dopaminergic manipulation affects motor cortical networks, and serotonergic manipulation affects both.

Finally, a number of studies have demonstrated that neuromodulators alter the topological properties of intrinsic activity correlations. These studies suggest a possible distinction between the effects of DA and NE on network-level functional integration. Similar to DA, 5HT seems to increase metrics of functional integration.

The studies discussed in this review may well have only scratched the surface the full spectrum of effects that neuromodulatory systems exert on intrinsic activity correlations. Even so, they open up exciting avenues for future work. An effort to map the contribution of each neuromodulatory system to the spatial and temporal features of intrinsic correlations may aid the identification of shared, independent, or antagonistic principles between the actions of different neuromodulatory systems and their diverse receptor classes. Such principles will inform models of healthy brain function and provide an important reference for the mechanistic understanding of neurological and psychiatric disorders. Ultimately, such principles may guide the way toward identification of specific molecular targets for mechanistically inspired and individualized pharmacological interventions in disorders of higher brain function. In what follows, we outline a number of important avenues for future research. Each of these could help advance our understanding of the principles that govern intrinsic brain dynamics and its dysfunctions in critical ways.

### Dissecting the Mechanisms of Brainstem Modulation of Cortical Correlations

We have highlighted that neuromodulatory brainstem systems may alter intrinsic correlations of cortical population activity through diverse mechanistic pathways. The fluctuating activity of brainstem nuclei may provide common drive to large swathes of cortical regions, or drive other subcortical regions (such as the pulvinar nucleus of the thalamus) that can in turn cause widespread changes in cortical activity (Nakajima and Halassa, 2017; Arcaro et al., 2018). Second, neuromodulatory brainstem systems may also modulate the cortical dynamic state, thereby altering correlations in cortical population activity indirectly. These two mechanisms are non-mutually exclusive: the physiological effect of any neuromodulatory action likely results from a complex mixture of both. Nevertheless, careful experimental manipulations should tease these mechanisms apart and provide insight into the consequences of each mechanism for intrinsic activity fluctuations within the cortex.

For example, in order to test whether neuromodulatory systems induce temporal correlations in the cortex through common drive, one can manipulate the *time varying* activity of brainstem nuclei, and examine the time varying signature of this manipulation in cortical activity. Using optogenetics, specific neuron types can be targeted such that non-neuromodulatory (e.g., GABAergic) long-range projections that emanate from these nuclei are not directly affected by the experimental manipulation. Moreover, electrophysiological recordings within the cortex would circumvent interpretational caveats that are inherent to the transformation of neural activity into the fMRI signal (Logothetis, 2008). Experiments of this kind have shown promise (Grandjean et al., 2019). Combining such manipulations with the administration of pharmacological blockade of ionotropic receptors could ultimately provide decisive evidence in favor of or against the notion of drive of intrinsic activity correlations by neuromodulators.

Additionally, seed-based correlation studies can provide evidence of co-fluctuating activity in brainstem neuromodulatory nuclei and the cortex in humans. In order to elucidate the relationship between each neuromodulatory system and correlated activity within the cortex, studies are needed in which activity in all nuclei is measured simultaneously, and the covariation between the individual nuclei is taken into account. Such analyses have not yet been conducted, but can be readily implemented with existing techniques.

Another possible means to distinguishing multiple mechanisms of action is to examine the spatial correspondence between the effect of a manipulation of neuromodulators on cortical intrinsic activity correlations, and the distribution of specific receptors. These comparisons are now possible using open-access databases of genetic expression of receptor types (Hawrylycz et al., 2012), validated for use in neuroimaging (Gryglewski et al., 2018). Such studies may benefit from analytical tools that are tailored to distil manipulation-related effects on cortical correlations in a specific direction, without relying on *a priori* selection of correlated networks (e.g., van den Brink et al., 2018a). Examining the spatial relationship between the effect of a manipulation and the receptors may also be indicative of whether the cortical effect of a neuromodulator is determined primarily by the anatomical projection profile of the nucleus that releases it, or if the receptor distributions weigh more heavily (Grandjean et al., 2019). Moreover, such analyses should be used to contrast the impact of ionotropic and metabotropic receptors.

### Developing Mechanistic Models and Biomarkers for Neuropsychiatric Disorders

A detailed understanding of how neuromodulators shape intrinsic activity correlations may aid the development of novel biomarkers for neurological and psychiatric disorders, as well as mechanistic models of these disorders. Several disorders are associated with dysfunctions in one or multiple neuromodulatory brainstem systems. For example, Parkinson’s disease is caused by degeneration of the dopaminergic midbrain nuclei, along with the noradrenergic LC. Cognitive decline in aging, in particular Alzheimer’s disease, coincides with degeneration of the cholinergic BF and possibly the LC. Major depression and schizophrenia are associated with disturbances in catecholaminergic and serotonergic systems.

These clear associations with neuromodulatory brainstem systems are currently not exploited for the early detection and classification of such disorders. In particular, the current classification and diagnosis of psychiatric disorders is solely based on subjective assessments of behavioral symptoms, irrespective of the underlying pathophysiological mechanisms (Insel et al., 2010; Krystal and State, 2014). One consequence of this coarse and phenomenological classification scheme is that patient populations in one diagnostic category are often heterogeneous in terms of the neural circuit deficits that give rise to the behavioral symptoms (Seaton et al., 2001; Insel et al., 2010). This hampers the development of individualized treatment plans that target the key circuit disorder that underlies the cognitive or behavioral deficits of a given patient.

The insight that neuromodulators profoundly shape intrinsic activity correlations that are evident with non-invasive neuroimaging techniques opens the door for overcoming these limitations in current clinical practice. The insight sets the stage for the development of neural markers of psychiatric disorders that are directly grounded in the underlying pathomechanisms and cortical signatures of neuromodulatory action. Specifically, the changes in correlation patterns associated with (manipulations of) a specific neuromodulatory system can provide a “reference template” to which alterations of correlation patterns associated with specific disorders can be compared. Such markers may prove to reflect an individual patient’s precise deficit more reliably and help identify molecular targets for pharmacological intervention.

In this light, it is important to evaluate the influence of behavioral context on the effect that neuromodulators exert on intrinsic activity correlations. Manipulation of neuromodulator levels has been shown to result in opposing effects on intrinsic activity correlations under different behavioral contexts (Coull et al., 1999; Shine et al., 2018b; Pfeffer et al., 2019). Direct comparisons of various neuromodulators within the same- and between different cognitive contexts can thus provide interpretational constraints on alterations of correlation patterns that are associated with psychiatric disorders. Moreover, such an approach may help resolve standing discrepancies in the literature regarding the direction of pharmacological effects on intrinsic activity correlations.

Lastly, neuromodulators interact, through reciprocal connections between the brainstem nuclei, shared cortical afferents, and cortical receptor co-expression. Because of this, dysfunction in a single neuromodulatory system is unlikely to occur without affecting others. Moreover, any neuromodulatory dysfunction that is associated with a psychiatric disorder may not be observable in intrinsic activity correlations as a linear summation of the above described reference templates of the individual neuromodulatory systems that are dysfunctional. Thus, further study on how the joint actions of neuromodulators shape cortical interactions is needed. A starting point is to incorporate receptor co-expression into large-scale computational models of cortical function, combined with coupling terms that link activity of one neuromodulatory system to that of another. This may capture interactions between these systems more accurately, and yield mechanistic insight about how dysfunction in these systems manifests itself at the level of intra-cortical processes and behavior.

## Author Contributions

RB and TD conceived the idea for this article. RB wrote the manuscript. RB, TP, and TD commented on and edited the manuscript.

## Conflict of Interest

The authors declare that the research was conducted in the absence of any commercial or financial relationships that could be construed as a potential conflict of interest.
